# Bite Force Analysis Using Numerical Simulation with Different Prosthesis Anchorages

**DOI:** 10.3390/biomimetics11100750

**Published:** 2026-10-18

**Authors:** Belén Lima-Sánchez, Álvaro-José Martínez-González, Jorge Trigo-Tirez, Pedro Gómez-Molina, Maribel González-Martín, Aida Gutiérrez-Corrales, Elena Oliva-Ferrusola, Eusebio Torres-Carranza, Alberto García-Perla-García, Gonzalo Ruiz-de-León, Daniel Torres-Lagares, María Baus-Domínguez

**Affiliations:** 1 Department of Stomatology, School of Dentistry, University of Seville, 41009 Seville, Spain; belima97@gmail.com (B.L.-S.); migm@us.es (M.G.-M.); agcorrales@us.es (A.G.-C.); eleolifer@alumn.us.es (E.O.-F.); ruizdeleong@gmail.com (G.R.-d.-L.); 2 ICEMM S.L.U., Engineering Specialized in FEM and CFD Simulation, C/Las Fábricas, 28923 Alcorcón, Spain; alvaro.martinez@icemm.es (Á.-J.M.-G.); jorge.trigo@icemm.es (J.T.-T.); pedro.gomez@icemm.es (P.G.-M.); 3 Oral and Maxillofacial Surgery Unit, Virgen del Rocío University Hospital, Avda. Manuel Siurot, 41013 Seville, Spain; etorres2@us.es (E.T.-C.); agarciaperla@us.es (A.G.-P.-G.)

**Keywords:** finite element analysis, bite force, occlusal force distribution, dental implants, zygomatic implants, implant-supported prostheses, biomechanics, maxillary rehabilitation, occlusion, numerical simulation

## Abstract

Background: Implant-supported prosthetic rehabilitation is widely used to restore function in edentulous patients. Although most implant finite element studies prescribe occlusal forces as input loads and evaluate the resulting stresses, the effect of implant-supported rehabilitation on bite-force distribution remains insufficiently characterized. This study evaluated how three clinically relevant implant-supported rehabilitation configurations modify occlusal force distribution relative to the physiological reference pattern. Methods: A three-dimensional finite element model of the head and neck, calibrated for physiological symmetric bite conditions, was developed using high-resolution anatomical reconstructions of the maxilla, mandible, zygomatic bones, and dentition. Three clinical configurations were analyzed: three conventional implants, one conventional plus one zygomatic implant, and two zygomatic implants. The model was calibrated to reproduce a total physiological occlusal force of 220.7 N, and the resulting force distribution was compared with published data for intact dentition. Results: All implant-supported configurations altered the physiological occlusal force pattern, concentrating between 71% and 78% of the total bite force on the first molars adjacent to the posterior implants (46–36). In contrast, the three implant configurations showed only minor differences in overall stress distribution in the maxillary and zygomatic bones. Conclusions: Implant-supported rehabilitation substantially modified the physiological bite-force distribution in all three simulated configurations, whereas only limited differences in the overall bone stress pattern were observed among the configurations. These differences should be interpreted at the configuration level, since the present study cannot separate the independent effects of implant number, position, and type design. In contrast, conventional or zygomatic implants have.

## 1. Introduction

The use of implants and prostheses to restore tooth loss has become common practice in contemporary dentistry. A wide range of implant and prosthesis types has been developed, evaluated, and brought to market to provide prosthetic, anatomical, aesthetic, and functional solutions for patients with partial or total tooth loss. Numerous implant systems are currently available, supported by the reliability demonstrated in a range of clinical studies.

The choice of implant type is generally determined by the patient’s bone quality and morphology. In contrast, the impact on the patient’s bite arising from the redistribution of occlusal loads between the molars is usually overlooked.

Finite element analysis (FEA) has become the reference tool for studying the biomechanical behavior of implant-supported restorations because it quantifies stresses and strains that are inaccessible to direct clinical measurement. Published work has focused mainly on the response of the peri-implant bone: the number and distribution of implants have been shown to govern prosthesis deformation and the uniformity of load transfer [1,2], the cervical region of the implant has been reported to concentrate the highest stress values systematically [3,4], and the superstructure material is particularly critical in zygomatic anchorages [5]. Specifically, the literature has compared the behavior of zygomatic implants with high-angulation all-on-four configurations [6]. Their usefulness has been confirmed in cases of severe maxillary atrophy, where conventional implants show clear limitations [7]. 

More recent FEA studies have extended these investigations to multiple conventional, zygomatic, and combined implant-supported configurations. Different numbers and arrangements of conventional and zygomatic implants have been evaluated [8], including comparisons under different prescribed loading conditions such as vertical and horizontal loads [9] and vertical and oblique loads [4]. Muscular loading has also been incorporated into craniofacial FEA of zygomatic rehabilitation, showing that inclusion of the muscle component can influence the predicted biomechanical response [10]. These studies have provided important information on how implant configuration and loading conditions affect stresses, strains, and displacements in the implant–prosthesis–bone system.

Despite these advances, in most implant FEA investigations, occlusal or prosthetic loads are prescribed as input boundary conditions, and the resulting stresses, strains, or displacements are evaluated as model outputs [1,2,3,4,5,6,8,10]. Consequently, these models provide valuable information on how a predefined loading condition is transferred through the implant–prosthesis–bone system. Still, they do not directly address how changes in the support conditions of the rehabilitated arch may alter the distribution of occlusal reaction forces generated during biting. This distinction defines the specific scientific gap addressed here: not the mechanical response of different implant configurations to a prescribed load but the relationship between implant-supported prosthetic support and the resulting distribution of bite force among occlusal contacts under a common muscular loading condition. This question is clinically relevant because occlusal loading is directly related to the biomechanical environment associated with marginal bone loss and peri-implant complications. However, all of these studies assess the response of bone and prosthetic components to an occlusal load [8,9,11,12].

The present study addresses this complementary biomechanical problem using the previously developed and calibrated head-and-neck FE model [13]. After calibration, muscular loading is maintained as the mechanical input producing mandibular closure. At the same time, the resulting forces transmitted at the posterior occlusal contacts are obtained as model outputs after replacing the natural maxillary support with different implant-supported prosthetic configurations. Thus, the purpose is not to introduce a new FE model or a new loading direction or merely to compare conventional and zygomatic implants but to determine whether the transition from physiological periodontal support to implant-supported prosthetic anchorage modifies the resulting posterior bite-force distribution and to characterize whether this redistribution differs among three implant-supported rehabilitation configurations.

Accordingly, we use the calibrated model to answer a different question: What happens to that distribution when the upper arch is rehabilitated with an implant-supported prosthesis, and does the chosen anchorage modify the outcome?

- Rehabilitation configuration 1: three conventional implants in positions 2, 4 and 6;
- Rehabilitation configuration 2: one conventional implant in position 3 and one zygomatic implant in position 6;
- Rehabilitation configuration 3: two zygomatic implants in positions 3 and 6.

Because these configurations differ simultaneously in the number, position, and type of implant anchorage, they were considered complete rehabilitation configurations rather than a controlled comparison designed to isolate the independent effect of conventional versus zygomatic implants. The evaluation method consisted of comparing the occlusal forces obtained at the three analyzed posterior pairs—namely, the second molars (47–37), the first molars (46–36), and the second premolars (45–35)—since these account for more than 95% of the occlusal force [14].

The starting hypothesis is that replacing physiological periodontal support with implant-supported prosthetic anchorage alters the posterior distribution of bite force and that the magnitude of this redistribution differs among rehabilitation configurations. The null hypothesis is that implant-supported rehabilitation does not appreciably modify the distribution obtained for intact dentition (conventional or zygomatic).

## 2. Materials and Methods

### 2.1. Definition of the FE Model

The baseline FE model [15] includes hundreds of materials, allowing the behavior of bone, tendons, muscles, and other soft tissues to be modeled accurately in both the elastic and plastic regimes, up to failure. However, that model does not provide sufficient detail to evaluate maxillary bone behavior, so we replaced these bones in the source model with others derived from real geometry obtained by 3D scanning [16].

The materials used for the mandible and the maxillary bones were defined based on the Misch classification [17,18] (Table 1), with type II bone, characterized by a cortical thickness of 1.5–2.0 mm, selected as the configuration.

### 2.2. Occlusal Forces

Under contact force between the upper and lower molars when no solid element is present, occlusal forces generated during biting result from the contraction of the jaw-closing muscles and the subsequent contact between the upper and lower dentition. Therefore, in this numerical framework, two distinct stages must be distinguished: calibration of muscular loading and prediction of occlusal force distribution in implant-supported configurations.

In the calibration stage, we approached inversely. Because the forces developed by the jaw muscles and tendons cannot be directly derived from the target bite-force distribution, reference occlusal reactions were prescribed at the tooth contacts, and the corresponding forces developed by the elements representing the jaw muscles and tendons were determined following the calibration procedure described by González-Martín et al. [13].

**Figure 1:**
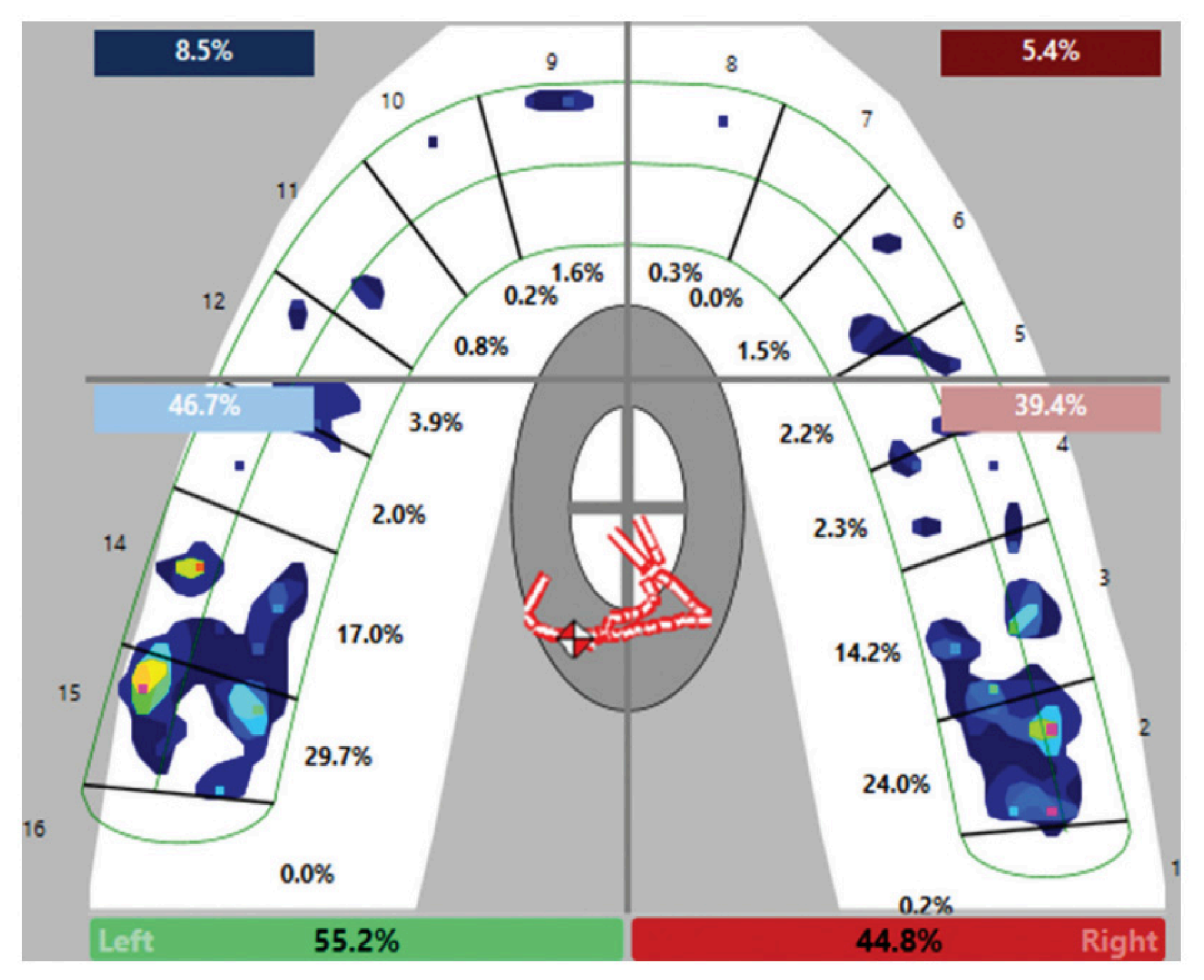
Distribution of bite pressures [11].

We derived the reference occlusal reactions from the studies of Turkistani et al. (2023) [14] and Nishigawa et al. (2001) [19]. Figure 1 shows the pressure distribution and percentage of force reported in [11]. For a total reference occlusal force of 220.7 N [16], the homogenized force values per tooth pair are given in Table 2.

The pressure values shown in Figure 1 for the incisors and canines were reassigned to the first molars, as they are marginal in relation to the forces exerted by the molars. This criterion is based on three reasons. First, the model does not include occlusal contacts in the anterior sector, so the load fraction corresponding to the incisors and canines must be transferred to one of the posterior pairs to preserve the total resultant of 220.7 N and its symmetry with respect to the mid-sagittal plane. Second, the first molar has the largest occlusal surface and the greatest physiological load capacity, and it is the usual reference for instrumental recording of bite force. Third, this choice raises the reference fraction attributed to the 46–36 pair, making the comparison with the implant-supported models more conservative, since the load migrates precisely toward that pair; any other distribution of the anterior fraction among the posterior pairs would increase the difference observed relative to intact dentition.

Once the muscular loading had been calibrated, the direction of the analysis was reversed for the implant-supported simulations. The calibrated muscular forces were maintained as input loads in each of the three clinical configurations, producing mandibular closure. In contrast, the forces generated at the posterior occlusal contacts were obtained as model outputs. Therefore, unlike conventional implant FEA approaches in which tooth loads are prescribed and the resulting stresses or strains are evaluated, the present simulations allowed the occlusal force distribution to emerge from the mechanical response of each prosthesis–anchorage configuration under the same calibrated muscular loading.

### 2.3. Finite Element Model

All analyses were performed using the finite element method with Abaqus Standard 2024 commercial software (Dassault Systèmes, Vélizy-Villacoublay, France). The analyses were solved as non-linear static problems with geometric non-linearity activated, using the bilinear pressure-overclosure contact law described in Section 2.5.3 and connectors without compressive stiffness for the musculature. Contact interaction was resolved using a surface-to-surface formulation with the penalty method, and the default Abaqus Standard convergence criteria for forces and displacements were adopted, with automatic incrementation.

The simulation model covers the head and neck down to the C7 cervical vertebra, retaining all soft tissues except the skin, eyes, and ears.

**Figure 2:**
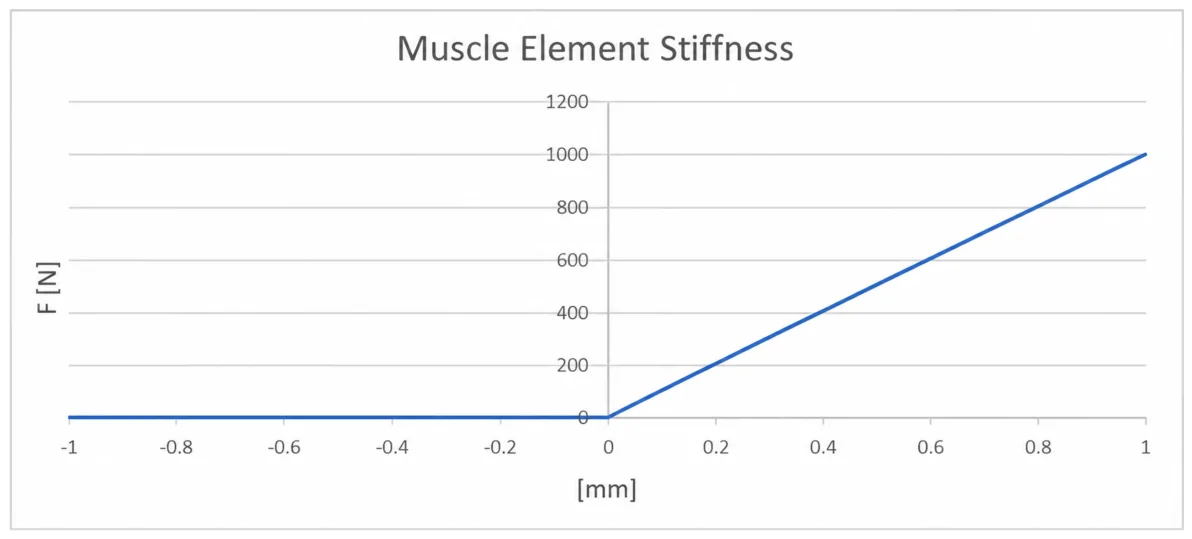
Stiffness of the elements modeling the temporalis and masseter muscles.

The elevator musculature was represented by the temporalis and masseter muscles, modeled using CONN3D2 connector elements. The connection between the connector and the bone was modeled with non-stiff distributing elements, so the connector load is spread over an area of influence and stress peaks in post-processing are mitigated. The behavior of these elements was defined by a constitutive law with no stiffness under compressive load and a tensile stiffness of 1000 N/mm. The following figure shows the constitutive law used in this study (Figure 2).

The element type used to model the maxilla, zygomatic bones, mandible, and teeth was the second-order tetrahedron (C3D10), with a mean mesh size of 1–1.5 mm [20]. The remaining parts of the model were derived from the original model according to [15,20], using first-order hexahedral C3D8 elements for the skull and first-order tetrahedral C3D4 elements for the vertebrae and complex-geometry parts.

As boundary conditions, all degrees of freedom were restrained at the nodes forming the base of the C7 vertebra.

The FE models of the different simulated rehabilitation configuration were created from the FE model of the intact configuration by modifying each bone affected by implant placement and prosthesis placement.

### 2.4. Clinical Rehabilitation Configuration Series

In all simulated rehabilitation configuration cases, the position of the implants is identified by the number of the corresponding tooth counted from the midline so that position 1 corresponds to the central incisor, 2 to the lateral incisor, 3 to the canine, 4 to the first premolar, 5 to the second premolar, and 6 to the first molar. The three configurations are symmetrical with respect to the mid-sagittal plane, so the total number of implants is twice the number indicated in each rehabilitation configuration case: six implants in simulated rehabilitation configuration 1 and four in cases 2 and 3.

#### 2.4.1. Simulated Rehabilitation Configuration 1

**Figure 3:**
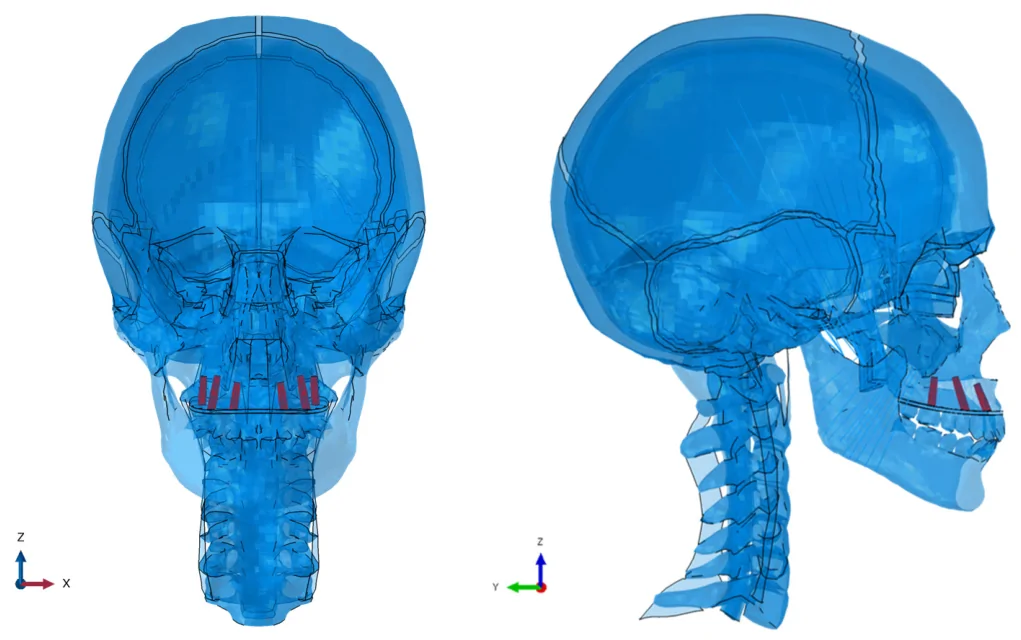
FE model. Frontal and lateral views of the simulated rehabilitation configuration for 1. The implants are highlighted in red, while the remaining components of the finite element model are shown in blue.

This model uses three conventional implants in positions 2, 4, and 6 (and their mirror images). The used implants are 13 mm in length and 4 mm in diameter (Figure 3).

#### 2.4.2. Simulated Rehabilitation Configuration 2

**Figure 4:**
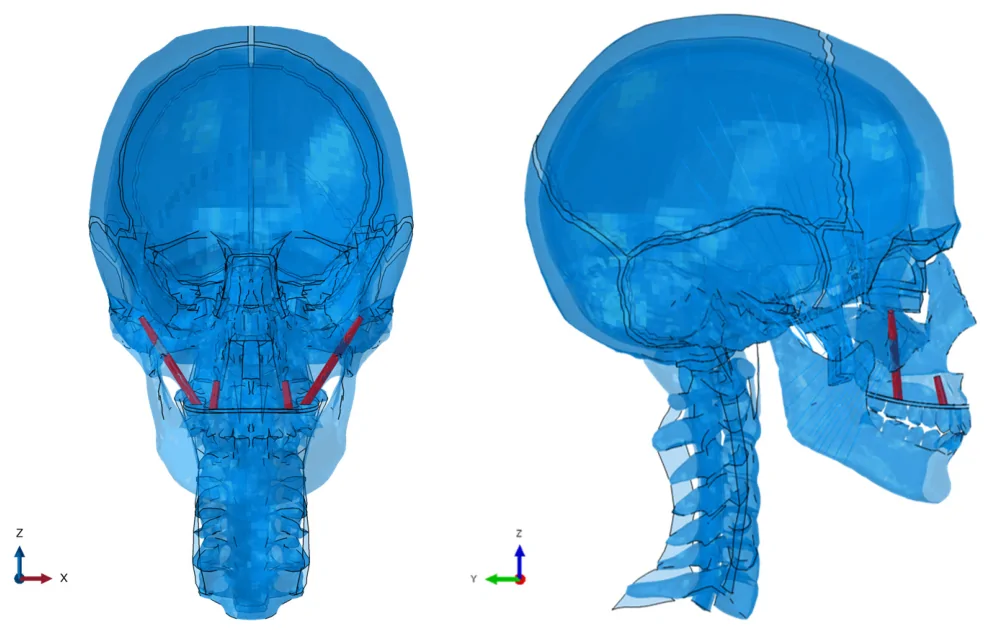
FE model. Frontal and lateral views of simulated rehabilitation configuration 2.

This model uses one conventional implant in position 3 and one zygomatic implant in position 6 (and their mirror images). The conventional implant is 13 mm long and 4 mm in diameter, and the zygomatic implant is 47.5 mm long and 4.2 mm in diameter (Figure 4).

#### 2.4.3. Simulated Rehabilitation Configuration 3

**Figure 5:**
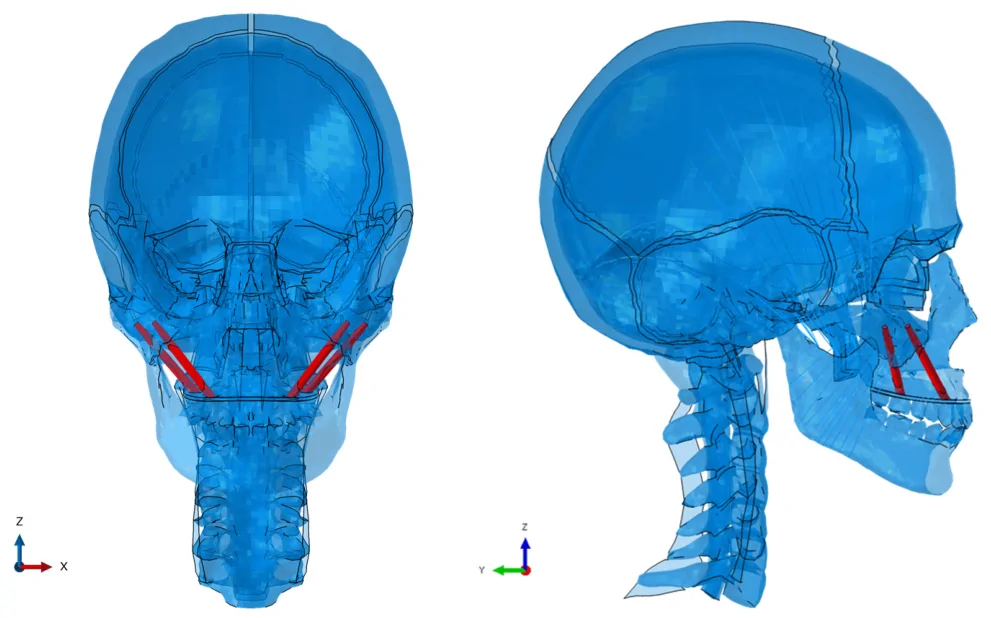
FE model. Frontal and lateral views of the simulated rehabilitation configuration for 3.

This model uses two zygomatic implants in positions 3 and 6 (and their mirror images). The implants are 47.5 mm long and 4.2 mm in diameter for the zygomatic implants in positions 3 and 4, and 40 mm long and 4.2 mm in diameter for the zygomatic implant in position 6 (Figure 5).

### 2.5. Constitutive Model

All materials were modeled as linear and isotropic, given the load level to which they are subjected.

#### 2.5.1. Interaction Between Teeth

Interaction between teeth occurs through contact between the individual units that make up the dentition. In an optimal configuration, the teeth are aligned so that contact between them is optimal. Representing this effect in simulation models is highly complex and requires very high mesh densities, along with defining contact stiffnesses between individual teeth, to accurately obtain contact forces. Because this effect is highly local and confined to the tooth surface, Saint-Venant’s principle allows for replacement of the contact interaction with an interaction based on flexible connectors that transmit the total contact force from one tooth to another.

**Figure 6:**
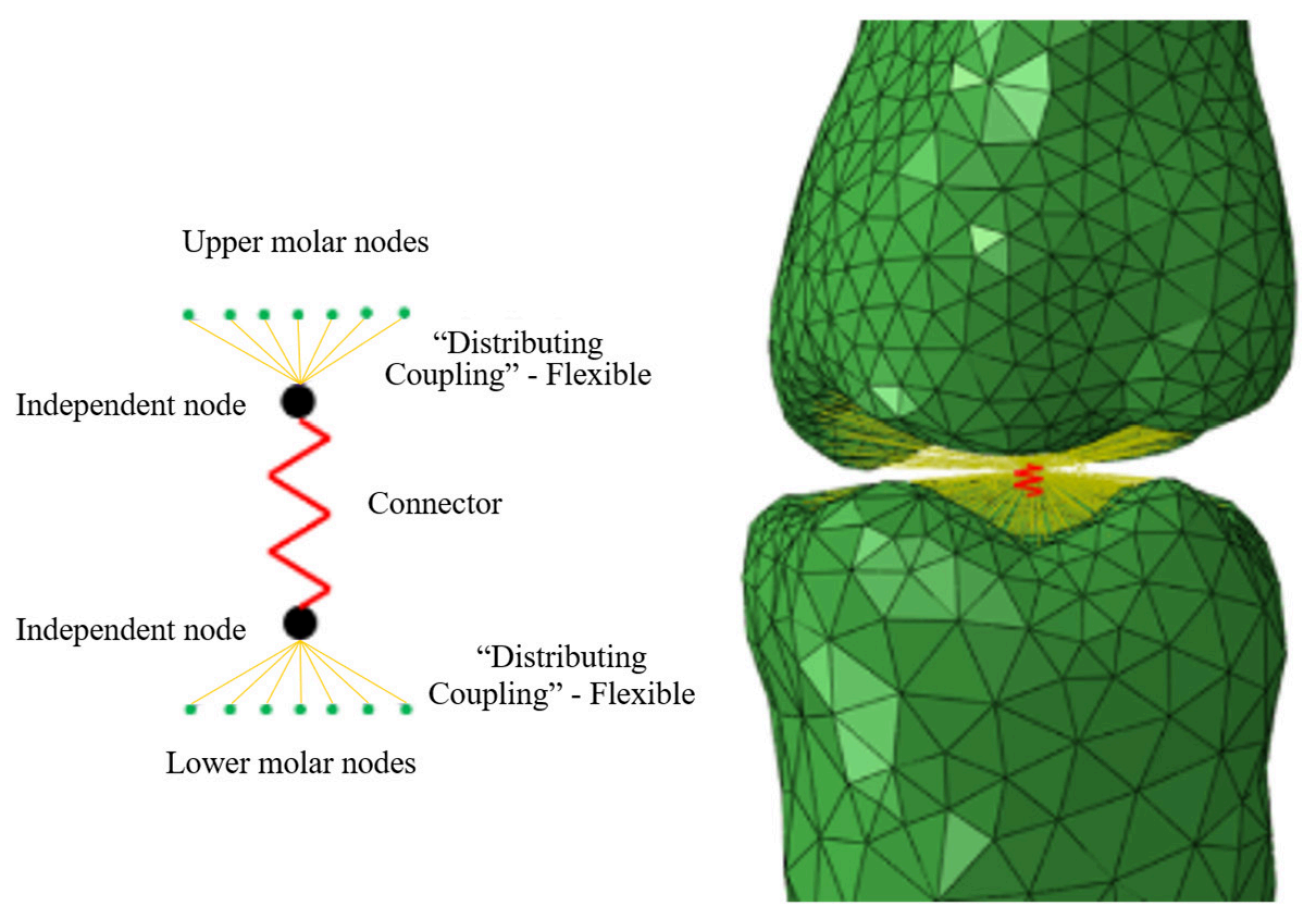
Model of the interaction between teeth.

The following image shows the modeling approach used to represent tooth contact (Figure 6).

The contact stiffness between the three pairs of teeth was obtained by an optimization process that minimized the following objective function:

(1) $$f=\sum_{i=1}^{3}\left|{CF}_{FEM,i}-{CF}_{Test,i}\right|,$$

where indices i = 1, 2, and 3 refer to the interaction between the 47–37, 46–36, and 45–35 tooth pairs; CF_FEM,i_ is the contact force obtained from the FE simulation, and CF_Test,i_ is the corresponding reference occlusal force for each tooth pair, as reported in Table 2 [13].

In the present study, we obtained contact stiffness for a single model representing the prosthesis without implants, rigidly bonded to the maxilla. We kept the value unchanged for the three studied simulated rehabilitation configurations.

#### 2.5.2. Interaction Between the Implants and the Maxillary and Zygomatic Bone

We established the connection between the implants and the maxillary and zygomatic bones using Tie kinematic constraints, which rigidly join the surfaces.

#### 2.5.3. Contact Interaction Between the Prosthesis and the Bone

We modeled normal contact interaction between bone and prosthesis using a bilinear constitutive law to account for the mucosa between the prosthesis and the implant. We therefore considered an allowable pressure of 0.1 N/mm^2^ for the initial compression up to 0.5 mm and 100 N/mm^2^ for the following 0.5 mm of compression. This definition accounts for a 0.5 mm thick mucosa with near-zero stiffness, followed by an interaction stiff enough to represent contact with the bone.

## 3. Results

After applying the process described in the previous section, we obtained the preload values in the jaw muscles for the baseline prosthesis model without implants, rigidly bonded to the maxilla.

**Figure 7:**
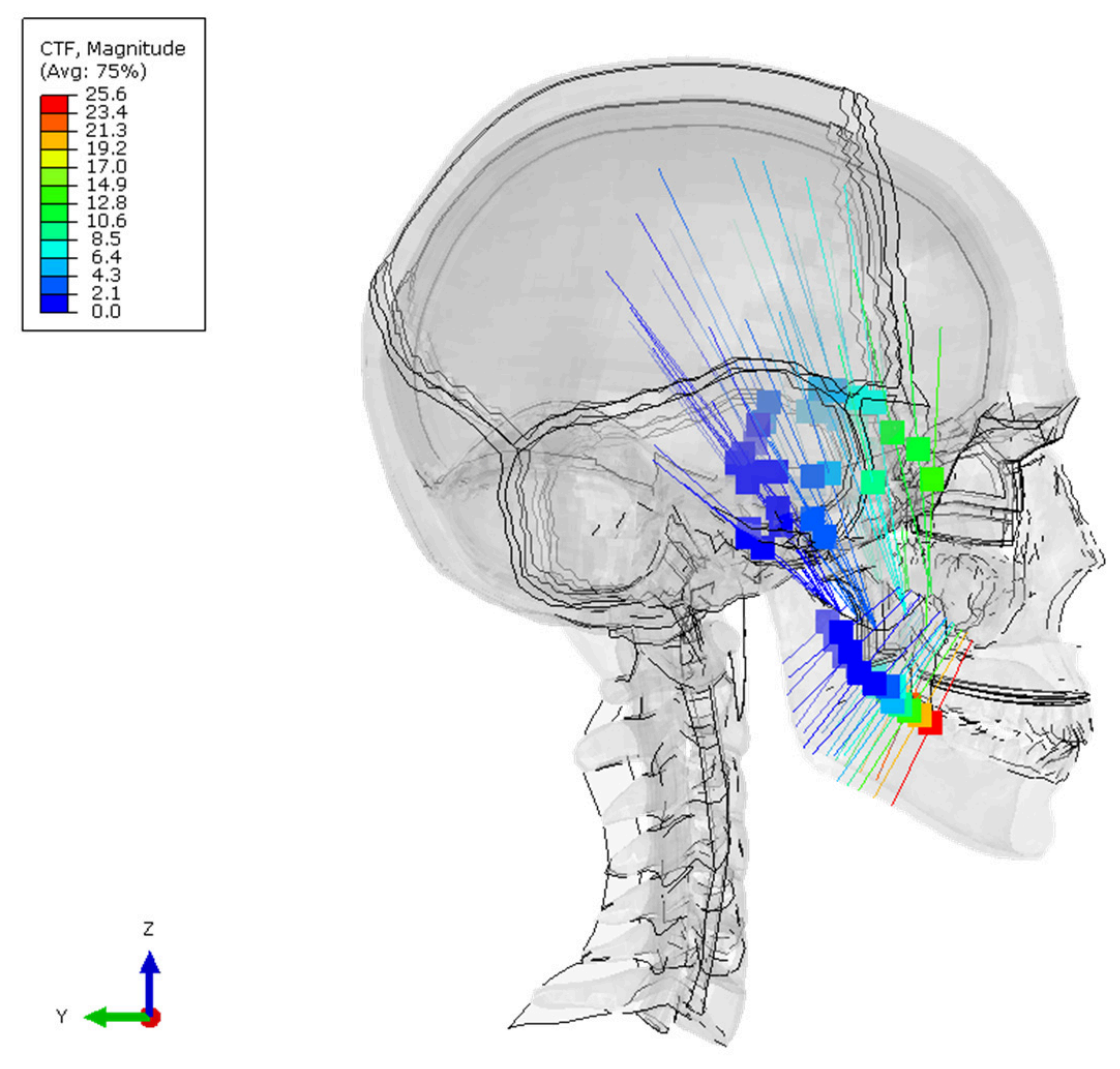
Forces exerted by the jaw muscles and tendons. Units [N].

Figure 7 shows the preload values, which are consistent with those obtained by González-Martín et al. (2025) [13].

**Figure 8:**
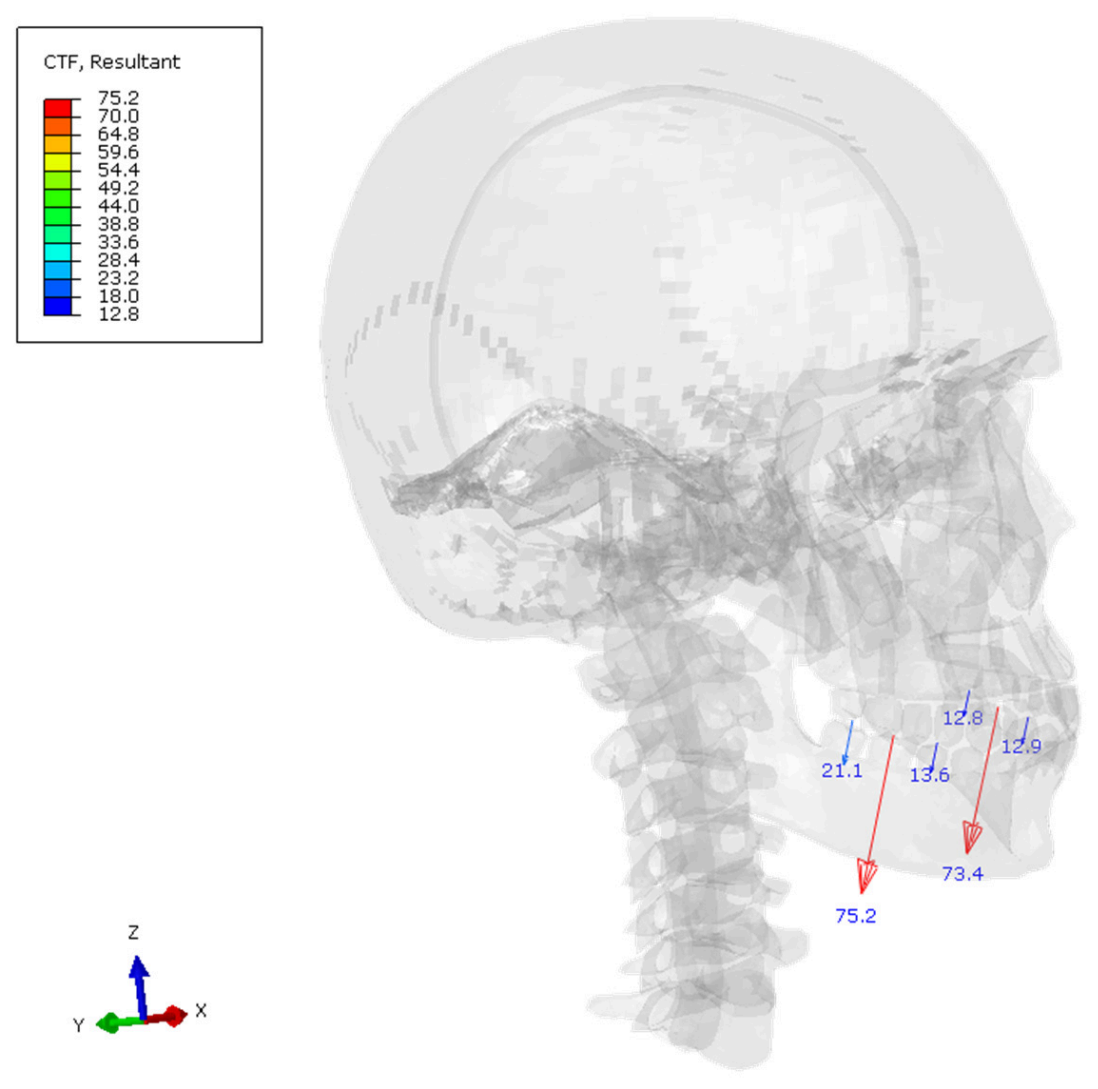
Occlusal forces for a 220.7 N bite—simulated rehabilitation configuration 1. Units [N].

**Figure 9:**
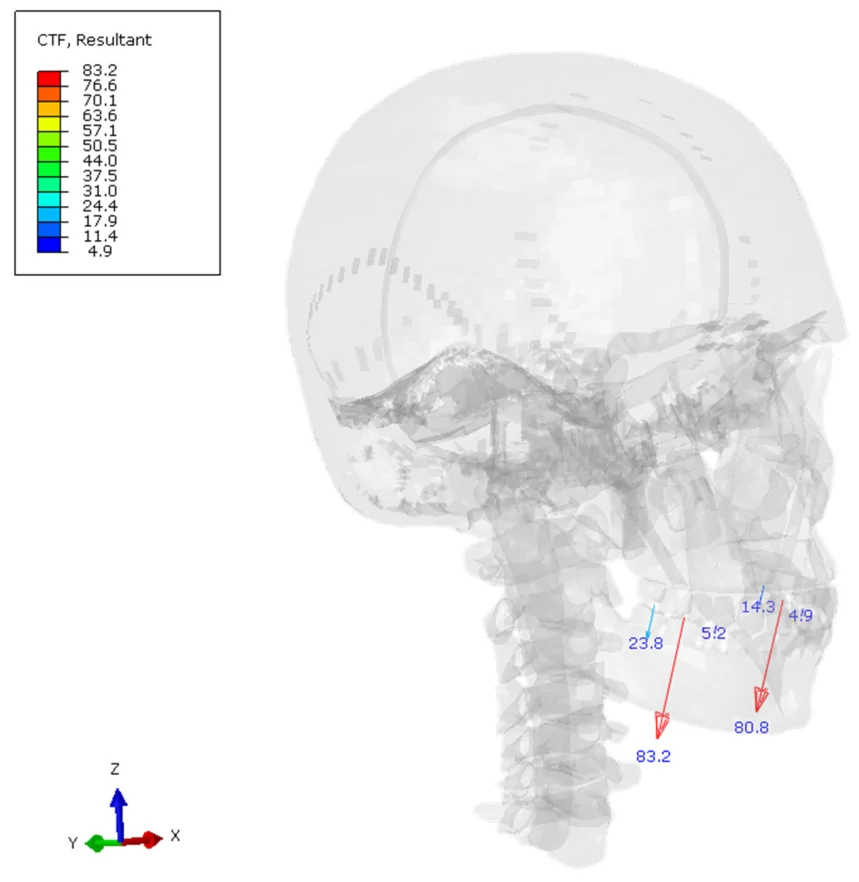
Occlusal forces for a 220.7 N bite—simulated rehabilitation configuration 2. Units [N].

**Figure 10:**
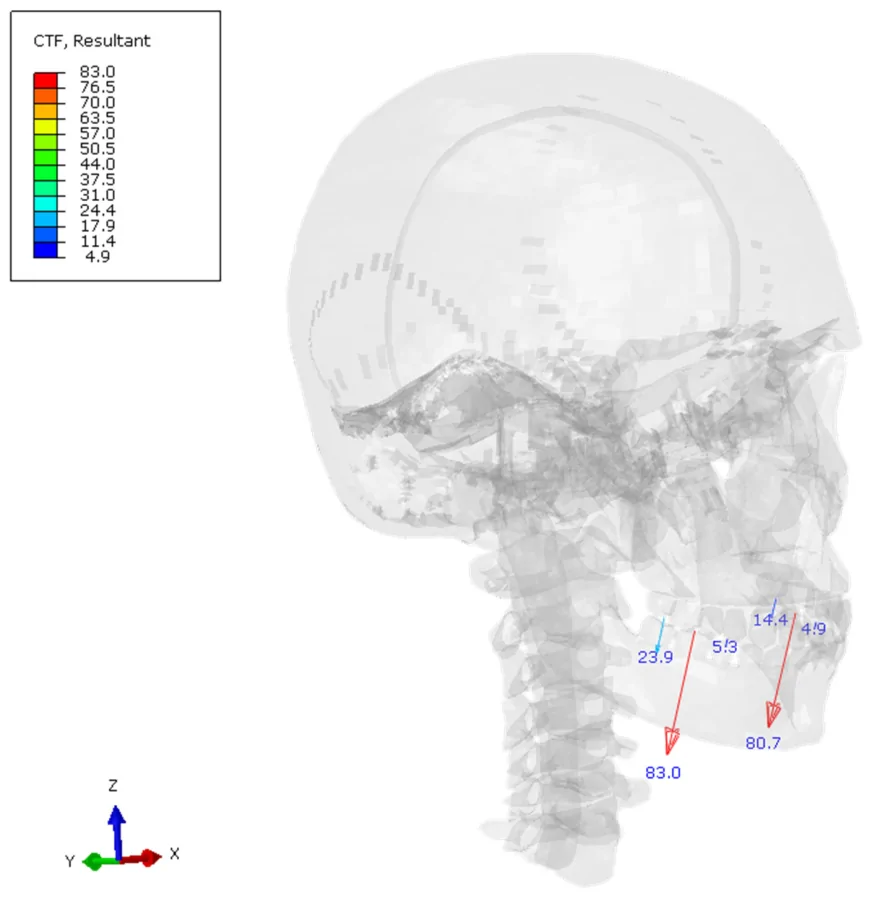
Occlusal forces for a 220.7 N bite—simulated rehabilitation configuration 3. Units [N].

Using the obtained preload values, the bite study was carried out for different simulated rehabilitation configurations. In each numerical model representing different prosthesis configurations, we introduced tensile forces on the jaw muscles. These forces close the mandible and, through tooth interaction, produce the occlusal force carried by each tooth. Figure 8, Figure 9 and Figure 10 show the occlusal force values obtained for each rehabilitation configuration in the clinical case.

The following table (Table 3) compares the occlusal forces from the FE models with the experimental reference values [14]. The model does not show perfect symmetry in the results, most likely because the mesh is not completely symmetrical, which may create interactions that are likewise not fully symmetrical and produce small variations such as those observed.

To compare the results with the experimental reference value, we paired the mean occlusal load for each tooth on the right side with its counterpart on the left.

**Figure 11:**
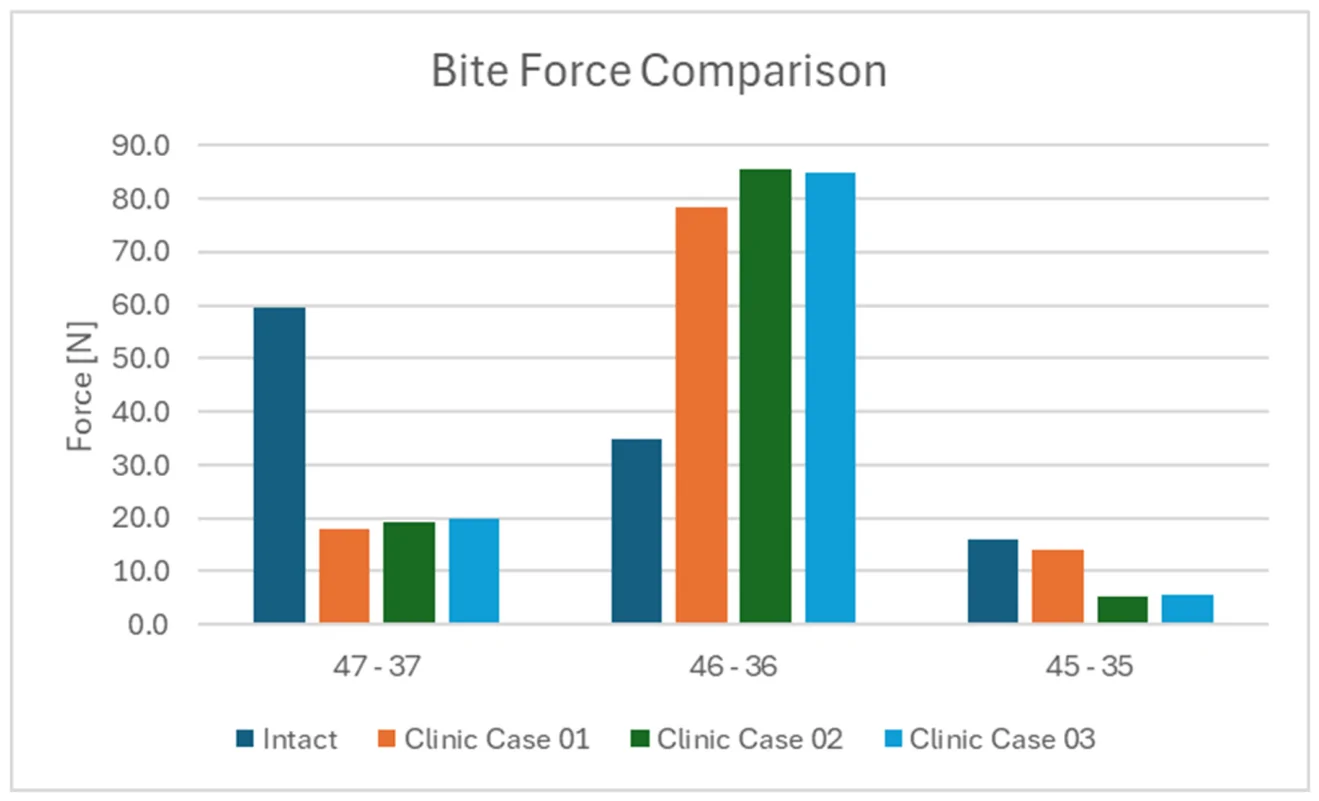
Comparison of the occlusal forces at the analyzed tooth pairs.

The following figure shows the load distribution across the tooth pairs graphically (Figure 11).

Modeling occurs in areas of interaction with the connectors modeling the jaw muscles, and results in these areas should be filtered.

**Figure 12:**
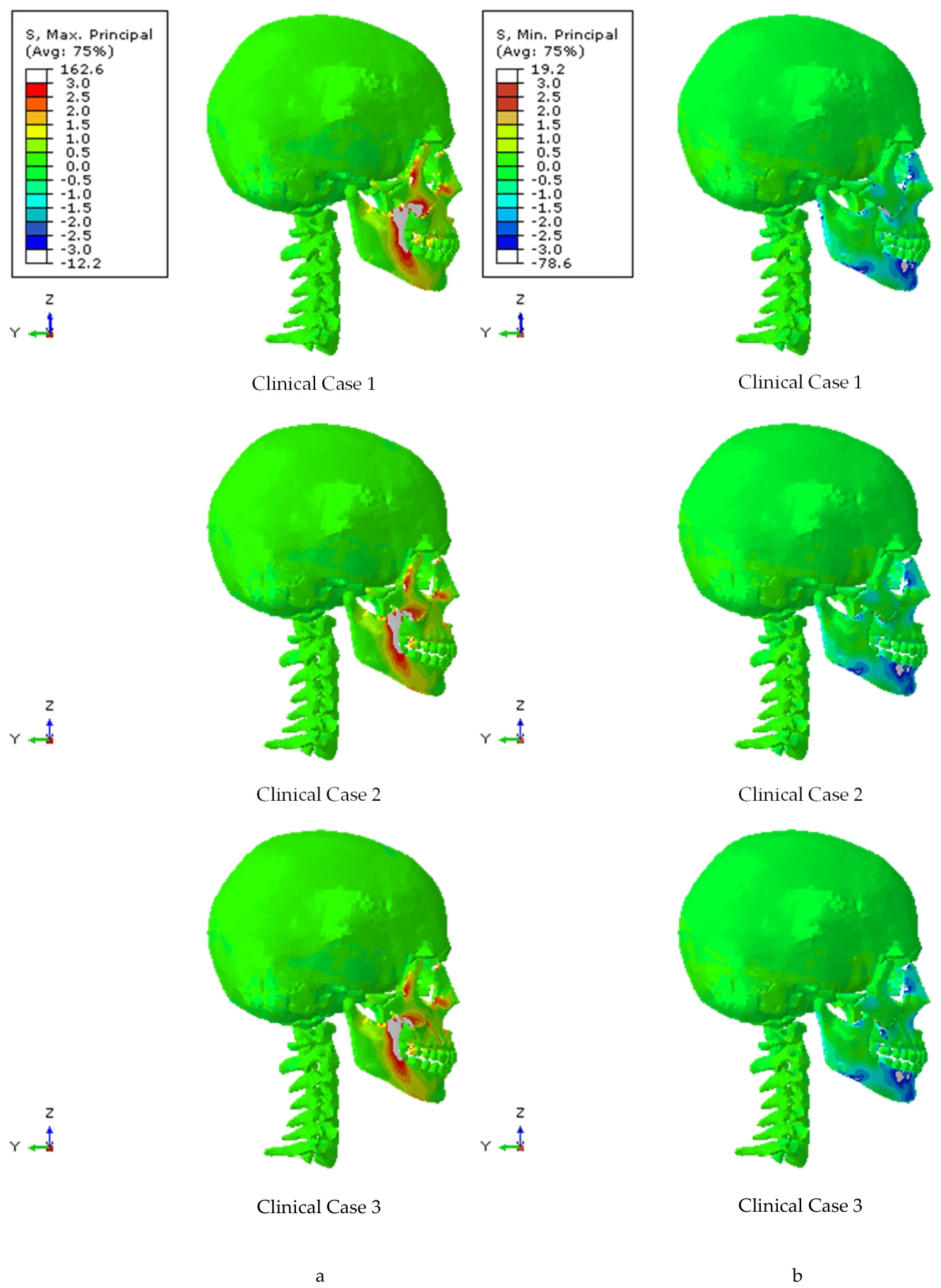
Stress state: (**a**) maximum principal stresses [N/mm^2^]; (**b**) minimum principal stresses [N/mm^2^].

**Figure 13:**
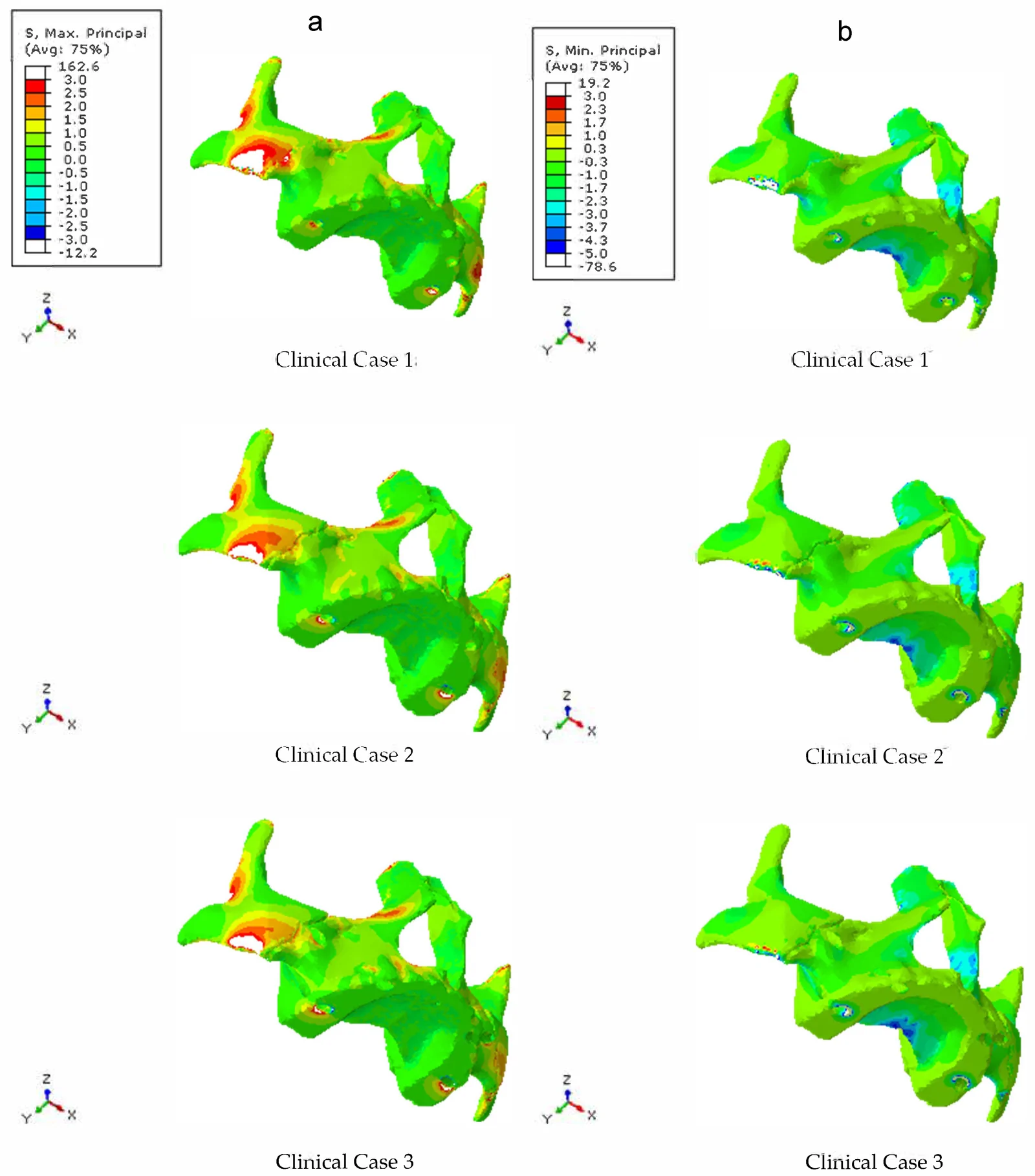
Stress state: (**a**) maximum principal stresses [N/mm^2^]; (**b**) minimum principal stresses [N/mm^2^].

The global and detailed maximum and minimum principal stress fields in the maxillary and zygomatic bones are shown in Figure 12 and Figure 13. The highest localized stress values were observed in regions where the connector elements representing the jaw muscles transfer loads to the three-dimensional bone mesh. These peaks are associated with numerical singularities generated by the connector-to-solid load-transfer formulation and were therefore excluded from the global bone stress patterns.

After identifying these connector-related singularities, we displayed the maximum and minimum principal stress fields using a +8/−4 MPa range to evaluate the broader stress distribution across the three rehabilitation configurations. Elements exceeding this range remain represented in the corresponding contour plots. In addition, the same stress fields are presented using a narrower ±3 MPa visualization range to provide greater resolution in regions away from the localized stress peaks. The ±3 MPa range is therefore used as an additional graphical representation and not as a criterion for excluding stress values or defining their clinical relevance.

## 4. Discussion

### 4.1. Alteration of the Physiological Bite Pattern by Implant-Supported Prostheses

Understanding occlusion in implant rehabilitation is essential because it reduces complications and improves patient satisfaction, as inadequate contacts cause instability in both dynamic and static occlusion [13].

In a healthy natural dentition, bite force distribution follows a clearly defined physiological pattern characterized by posterior predominance and a balanced distribution among the molars. Clinical studies based on dynamic occlusal records show that molars bear three to six times as much load as anterior teeth while maintaining bilateral symmetry. Turkistani et al. (2023) [14] analyzed the relative distribution of bite force in subjects with a complete permanent dentition. They observed that, in individuals with class I occlusion, occlusal load progressively concentrates in the posterior region, without maximum load concentrating on specific teeth.

This physiological pattern is interpreted as a decreasing triangular distribution of the posterior load, in which the distal molars bear the greatest proportion of the force, followed by the mesial molars and, finally, the premolars. This arrangement allows occlusal loads to be organized efficiently along the arch.

The findings of Turkistani et al. (2023) [14] are consistent with those of Nishigawa et al. (2001) [19], who analyzed occlusal force distribution in natural dentition through repeated quantitative recordings. They described a physiological pattern of hierarchical posterior distribution in which occlusal load decreases progressively from the distal molars toward the premolars, creating a stable functional model reproducible across subjects with intact dentition. This pattern is characterized by higher load on teeth 47–37, followed by 46–36 and, finally, 45–35. It reflects an efficient biomechanical adaptation of the masticatory system, in which force is gradually dissipated through the periodontal ligament and alveolar bone [19]. It reflects an efficient biomechanical adaptation of the masticatory system, in which force is gradually dissipated through the periodontal ligament and alveolar bone [19].

More recent clinical T-Scan studies have further shown that individual occlusal characteristics influence posterior occlusal force distribution and may change following restorative treatment. Abutayyem et al. (2023) reported differences in molar force percentages among subjects with different malocclusion patterns, supporting the influence of occlusal configuration on force distribution [21]. Similarly, Chan et al. (2024) observed changes in molar occlusal force distribution following fixed prosthetic restoration, highlighting the adaptive and time-dependent nature of the occlusal response [22].

Nishigawa et al. (2001) [19] further noted that this pattern does not depend exclusively on the absolute magnitude of the bite force but on the adaptive capacity of the tooth–periodontal ligament–bone complex, which allows for small micromovements and a continuous redistribution of loads during occlusal contact. This is why, even under high load conditions, the natural dentition maintains a functional organization that avoids localized stress concentrations on individual teeth.

The results of the present study show that this physiological pattern is altered in the presence of implant-supported prostheses. In the analyzed models, the decreasing triangular distribution observed in the intact dentition breaks down. The load maximum migrates toward the 46–36 region, where 71% to 78% of the total applied load is concentrated, depending on the configuration (71.1% with three conventional implants, 77.6% with one conventional and one zygomatic implant, and 77.0% with two zygomatic implants). This redistribution contrasts with the findings of Turkistani et al. (2023) [14] and Nishigawa et al. (2001) [19], where even under non-ideal occlusal conditions, such as a class III occlusion, posterior load is not accompanied by overload of a single tooth or region.

### 4.2. Influence of the Stiffness of the Prosthesis–Implant System on Load Redistribution

The altered physiological bite pattern observed in the implant-supported models may be explained, at least in part, by differences in mechanical stiffness between the components of the prosthesis–implant system and the natural dentition. Unlike the tooth–periodontal ligament–bone complex, which is characterized by viscoelastic and adaptive behavior, the implant-supported system introduces structurally rigid elements that modify the transmission and redistribution of occlusal loads.

From a structural perspective, the implant-supported prosthesis behaves as a relatively flexible element, particularly when it extends distally or has regions with less implant support. FE studies show that this flexibility leads to prosthesis bending, which alters the homogeneous distribution of loads and favors stress concentration in specific regions of the system [1]. Aguir et al. (2023) observed that models with fewer implants show greater prosthesis deformation and stresses, even under symmetrical vertical loads [1].

In addition to this structural flexibility, the mucosa acts as a deformable interface that introduces differential displacements between the prosthesis and the bone. Although the mucosa can partially absorb the load, its elastic behavior allows the prosthesis to tilt and progressively transfer load towards the stiffer points of the system. This phenomenon has been widely described in numerical models of implant-supported complete prostheses, where soft tissue deformability contributes to non-uniform force transmission [2].

Under these conditions, implants act as preferential rigid load transmission paths, channeling forces directly to the peri-implant bone. Anitua et al. (2023) showed, using finite element analysis, that regions near the implants concentrate the highest stress and strain values, regardless of the total number of implants, and that this concentration intensifies with prosthetic extensions or cantilevers [2]. Consistent with these findings, Aguir et al. (2023) observed that most stress is located in the cervical region of the implants, confirming their role as structurally rigid elements within the system [1].

This interpretation is consistent with previous finite element investigations showing that load transfer within implant-supported rehabilitations depends not only on implant anchorage but also on prosthetic design and loading direction. Zupancic Cepic et al. (2022) observed that implant number, implant length, prosthetic splinting, and axial or oblique loading substantially influenced the stress distribution within the implant–prosthesis–bone system [23]. Similarly, Dario et al. (2023) reported differences in stress transfer between parallel and tilted All-on-Four configurations, with the biomechanical response also being influenced by cantilever length and mandibular anatomy [24]. Although these studies did not evaluate occlusal force distribution as a model output, their findings support the concept that the structural characteristics of the rehabilitation modify the pathways through which occlusal loads are transmitted [23,24].

The results of the present study fit within this biomechanical framework. The maximum load concentration in the molar region adjacent to the implants, reaching 71% to 78% of the total load, supports the system’s tendency to redistribute forces toward the stiffest regions. This behavior may relate to the interaction between prosthesis flexibility, mucosal deformability, and the high stiffness of the implants.

Nevertheless, the magnitude of the observed load concentration should be interpreted in light of the model assumptions. Previous finite element studies have shown that assuming linear, homogeneous, and isotropic bone properties does not fully reproduce bone’s complex, anisotropic behavior. Isotropic bone properties do not fully reproduce the complex and anisotropic behavior of real bone and may therefore influence the predicted biomechanical response [2]. Likewise, the rigid implant–bone interface used in the present model represents an ideal condition of complete osseointegration and does not reproduce micromotion, progressive healing, or bone remodeling. These assumptions may affect the effective stiffness of the implant-supported system and, consequently, the predicted stress and occlusal force distributions. However, because the same material properties and interface conditions were applied to all three implant-supported configurations, the model allows for comparison of their relative biomechanical behavior under standardized conditions [2]. Therefore, the 71–78% load concentration observed at the first molars should be interpreted as a model-dependent estimate indicating a clear tendency toward load localization, rather than an exact value directly transferable to every clinical situation.

### 4.3. Biomechanical Comparison Between Conventional and Zygomatic Implants

Load distribution changes with the anchorage used configuration; this does not substantially alter how zygomatic implants could reduce bone’s global stress state. However, the three configurations differ in the number and position of implants. High-load conditions and material fatigue phenomena do not appear to compromise the integrity of the system; critically, they improve load distribution and reduce prosthetic material choice, since the three implant-supported configurations differed in occlusal force distribution but showed relatively similar global stress patterns in the maxillary and zygomatic bones. Previous finite element studies have shown that the biomechanical response of implant-supported rehabilitations depends on several interacting factors, including implant number and position, implant inclination, prosthetic design, and superstructure material [1,2,3,4,5,6]. In zygomatic rehabilitation specifically, Tezerişener et al. (2024) [6] reported differences in cortical bone loading compared with high-angulation all-on-four configurations. In contrast, Gümrükçü et al. (2024) [3] showed that combining zygomatic implants with anterior conventional implants may improve load distribution and reduce prosthetic displacement. These findings highlight the strong dependence of the biomechanical response on the overall rehabilitation configuration rather than on a single design variable.

In the present study, simulated rehabilitation configuration 1 (three conventional implants per side) showed the most uniform occlusal force distribution among the three implant-supported configurations, with 71.1% of the total load concentrated at the 46–36 pair, compared with 77.6% in simulated rehabilitation configuration 2 (one conventional and one zygomatic implant per side) and 77.0% in simulated rehabilitation configuration 3 (two zygomatic implants per side). However, these differences cannot be attributed specifically to implant type. The three configurations differ simultaneously in the number of anchorages (six implants in simulated rehabilitation configuration 1 versus four in clinical simulated rehabilitation configurations 2 and 3), their positions along the arch, and the use of conventional and/or zygomatic anchorage. Consequently, the present study allows for comparison of the biomechanical response of the complete rehabilitation configurations. However, it does not allow for isolation of the independent effects of implant number, position, and type.

This distinction is particularly relevant when interpreting the relatively similar global bone stress patterns observed across the three models. The present findings indicate that, under the simulated conditions, changing the complete rehabilitation configuration produced only limited differences in the global stress state of the maxillary and zygomatic bones. They should not, however, be interpreted as evidence that conventional and zygomatic implants have equivalent biomechanical effects, since implant type was not varied independently. The remaining design parameters were held constant.

The literature nevertheless provides relevant context for the behavior of the individual components included in these configurations. Gümrükçü et al. (2024) [3] and Deniz et al. (2025) [4] identified the implant neck as a region of high stress concentration, reinforcing its importance as a critical area from a biomechanical perspective. In addition, Heboyan et al. (2022) [5] highlighted the influence of prosthetic material on the biomechanical response of zygomatic implant-supported rehabilitations, while the anchorage of zygomatic implants in the dense zygomatic bone provides a rehabilitation option in situations of severe maxillary atrophy in which conventional implant placement may be limited [7].

A controlled parametric analysis would be required to determine the independent contribution of implant type to occlusal force redistribution and bone stress. Such an analysis should maintain implant number; prosthetic geometry; loading conditions; and, as far as anatomically feasible, implant position while varying the anchorage type. The present results should therefore be regarded as a comparison of three clinically relevant rehabilitation configurations and as a basis for designing future controlled numerical studies aimed at separating the contributions of individual anchorage variables.

### 4.4. Clinical Implications of Bite Force Redistribution

The obtained results suggest that rehabilitation with implant-supported prostheses does not reproduce the physiological force distribution pattern characteristic of the natural dentition but, instead, induces a significant load concentration on the molars adjacent to the posterior implants. This redistribution is particularly clinically relevant because localized overload is one of the main biomechanical factors associated with biological and mechanical complications in implant-supported rehabilitations.

Unlike natural teeth, implants lack a periodontal ligament and associated proprioceptive mechanisms, which considerably reduces the system’s capacity to damp and redistribute occlusal loads. As a result, forces are transmitted directly to the peri-implant bone, favoring local stress and strain concentrations. Berzaghi et al. (2025) emphasize that this lack of physiological damping makes implants more susceptible to occlusal overload, especially in complete implant-supported rehabilitations, where biomechanical stability depends largely on occlusal design [11]. This emphasizes that this absence of physiological damping makes implants more susceptible to occlusal overload, especially in complete implant-supported rehabilitations, where biomechanical stability depends largely on occlusal design and contact distribution [11].

The clinical relevance of these load concentrations is widely documented. In a recent systematic review, Mojaver et al. (2025) concluded that excessive occlusal forces generate non-physiological stress distributions around implants and peri-implantitis [12]. The authors describe bone losses associated with occlusal factors. In contrast, traumatic overload can produce bone changes of between 1 and 3 mm and increase the incidence of peri-implantitis to values of between 20% and 0.65 to 1.20 mm. In contrast, traumatic overload can produce bone changes of 1 to 3 mm and increase the incidence of peri-implantitis to 20% to 50%. They also emphasize that mechanical overload combined with bacterial inflammation can significantly accelerate deterioration of peri-implant tissues [11,12].

These findings are consistent with the present study, where a bite load of 71–78% of the total load on the 46–36 molars could create a less favorable biomechanical environment than that observed in the natural dentition. Pérez-Pevida et al. (2021) showed that peri-implant bone loss significantly modifies the magnitude and distribution of the stresses transferred to the supporting bone, particularly increasing strains in the trabecular bone as marginal bone loss progresses [25]. The authors suggest a bidirectional relationship between overload and bone resorption: bone loss increases transmitted stresses, which may, in turn, favor the progression of peri-implant damage [25].

From a prosthetic perspective, non-physiological load redistribution may also favor mechanical complications. Several studies have linked localized stress increases to a higher risk of screw loosening, fracture of prosthetic components, fracture of the ceramic veneer, and even implant failure. Younesi et al. (2024) observed the effects of minimizing load concentrations [19,26].

The obtained results also have direct implications for surgical and prosthetic planning. The load concentration observed around the posterior implants supports the classical recommendation to maximize distal support by appropriately distributing the number and position of implants. Although no absolute consensus exists on an ideal configuration, many authors agree that increasing the number of implants and reducing the distances between supports lowers stresses transmitted to the peri-implant bone and prosthetic components. In this respect, Younesi et al. (2024) showed that increasing the number of teeth and implants significantly reduces stresses in the prosthetic system and in the supporting bone [26].

A particularly important consideration is the presence of distal cantilevers. Biomechanically, cantilevers increase the lever arm of occlusal forces and promote bending moments on the terminal implants. Berzaghi et al. (2025) note that the distal extension of the prosthesis is a major biomechanical risk factor in complete implant-supported rehabilitation and recommend limiting its length to the clinically necessary minimum to reduce loads transmitted to the most distal implants [11]. The results of the present study therefore support strategies aimed at minimizing or avoiding extensive cantilevers whenever anatomy and available bone allow.

Likewise, prosthetic design and occlusal adjustment are important. The current literature agrees that a balanced distribution of occlusal contacts and the elimination of eccentric interferences help to reduce local stress concentrations. Mojaver et al. (2025) emphasize that appropriate occlusal management is one of the most important factors in minimizing marginal bone loss associated with overload, while Berzaghi et al. (2025) recommend individualizing the occlusal scheme according to each patient’s biomechanical risk and the specific characteristics of the rehabilitation [11,12].

Finally, the obtained results allow the role of zygomatic implants to be interpreted from a biomechanical perspective. Although these implants represent a predictable and effective solution in patients with severe maxillary atrophy, the analyzed models indicate that they do not eliminate the load redistribution phenomenon observed in implant-supported prostheses. Instead, they shift the force transmission path toward anatomical regions with greater bone availability and density. Zygomatic implants should therefore be understood as a strategy for providing structural support in situations of compromised bone, not as a mechanism that can restore the physiological force distribution pattern of the natural dentition. Consequently, the same principles of occlusal control, prosthetic design optimization, and overload limitation remain applicable in this type of rehabilitation.

### 4.5. Study Limitations

The results of this study must be interpreted in the light of the limitations inherent to finite element analysis. Although this methodology is a widely validated tool for studying force and stress distribution in complex biomechanical systems, the results necessarily depend on the assumptions adopted during model construction. González-Martín et al. (2025) emphasized that FE model accuracy depends on the geometric definition, the mechanical properties assigned to the materials, and the loading conditions used [13].

One limitation of this study is that we assumed all materials were linear, homogeneous, and isotropic. However, cortical and trabecular bone has more complex mechanical properties that vary with internal structure, loading direction, and time [4,5,13]. Therefore, the model may not fully reproduce how the maxillary and zygomatic bones deform and transmit forces under clinical conditions. Although this simplification is common in finite element studies and allows for comparison across configurations under the same conditions, it may affect predicted bone stresses and occlusal force distribution. Accordingly, the 71–78% load concentration observed at the first molars should be considered a model-dependent estimate rather than an exact clinical value. Without patient-specific material properties and additional sensitivity analyses, we cannot determine the precise influence of this assumption.

The model also assumed perfect osseointegration of tie constraints between the implants and the surrounding bone. This formulation represents a completely bonded and mechanically stable interface and therefore prevents relative displacement between the implant and bone. Consequently, it does not allow for assessment of implant micromotion, progressive osseointegration, bone remodeling, or partial loss of integration [5]. By representing the implant–bone interface as ideally rigid, the model may overestimate the stiffness of the implant-supported load path and alter both peri-implant stress values and the proportion of occlusal load transferred towards the regions adjacent to the implants. This assumption may therefore have contributed to the predicted 71–78% load concentration at the first molars. However, the present simulations cannot establish the magnitude and direction of this influence. Thus, the results should be interpreted as representing an idealized condition of complete osseointegration rather than the full range of clinical interface conditions.

Another important limitation is that the analysis was carried out only on Misch type II bone. Bone quality significantly influences the stress distribution around implants, so these results cannot be extrapolated directly to type III or type IV bone, which is frequent in patients with severe maxillary atrophy [1]. This aspect is particularly relevant in rehabilitations with zygomatic implants, where the quality and quantity of available bone are usually compromised. In particular, the finding of a similar global bone stress state across the three configurations is limited to the simulated type II bone conditions. It should not be extrapolated to atrophic maxillae with poorer bone quality.

The loading conditions constitute an important limitation of the present study. The model simulated a single static and symmetric bite condition corresponding to a total reference occlusal force of 220.7 N. This standardized condition was selected to provide a common reference for comparing the mechanical responses of the different rehabilitation configurations rather than to reproduce the full complexity of functional mastication. In vivo mastication is dynamic and frequently asymmetric, involving unilateral contacts and force components of varying magnitude and direction, including oblique and lateral loading. Previous finite element investigations support the relevance of this limitation. Zupancic Cepic et al. (2022) demonstrated that axial and oblique loading produce different biomechanical responses in implant-supported restorations, while Dario et al. (2023) showed that the stress response of parallel and tilted implant configurations varies under inclined loading [23,24]. In addition, parafunctional conditions such as bruxism may generate loads considerably higher than those occurring during normal function and may modify stress distribution within the implant–prosthesis–bone system. The loading conditions are also a simplification of real functional behavior [12,19]. Loading direction is particularly relevant when interpreting zygomatic implant-supported configurations because of the long and inclined geometry of these implants and the resulting sensitivity of their biomechanical response to non-axial loading [4].

Consequently, the occlusal force redistribution predicted in the present study should be interpreted as specific to the investigated static and symmetric loading condition. It cannot be directly extrapolated to functional mastication. In particular, the present simulations cannot determine whether the 71–78% concentration of the analyzed posterior occlusal force at the first molar pair persists, increases, decreases, or shifts toward other contacts under asymmetric, unilateral, oblique, lateral, or dynamic loading. Therefore, this finding should be regarded as a model-dependent response under the specific loading condition analyzed rather than as a general prediction of occlusal force distribution during clinical mastication.

The elevator musculature includes the temporalis and masseter muscles. The medial pterygoid, which also participates in mandibular closure, and the suprahyoid musculature were not included. This simplification is common in static symmetrical bite models, in which the contribution of the medial pterygoid acts in a direction close to that of the masseter and is partially absorbed by the inverse calibration of the muscle preload described in Section 2.2. It may nevertheless affect the local orientation of the loads transmitted to the maxilla and limits the extrapolation of the results to lateral or eccentric movements.

Furthermore, we represented the occlusal interaction with equivalent connectors instead of real three-dimensional tooth contacts. This strategy simplified the model and reduced computational cost while maintaining a global force distribution consistent with previous studies. This approach does not, however, reproduce local clinical phenomena such as premature contacts, occlusal interferences, or individual anatomical variations [13].

A fundamental limitation of the present study concerns the distinction between model calibration and independent validation. We calibrated the contact stiffness between teeth using a single baseline model in which the prosthesis was rigidly bonded to the maxilla, based on occlusal force-distribution data reported for intact dentition. We then maintained this calibrated contact stiffness unchanged across the three implant-supported configurations. This approach ensured methodological consistency and provided a common physiological reference for exploring the relative influence of the different prosthetic support configurations under identical contact conditions. However, calibration against intact-dentition data does not constitute independent validation of the predictions obtained for the implant-supported configurations. No independent in vitro or clinical measurements of occlusal force distribution were available for any of the three implant-supported configurations; we could not experimentally verify the validity of this stiffness parameter or interpret the comparison between the implant-supported configurations and intact dentition.

Consequently, the predicted 71–78% concentration of the analyzed posterior occlusal force at the first molar pair (46–36) should be interpreted as a model-dependent numerical prediction under the specific assumptions, calibrated contact parameters, and investigated loading conditions rather than as an experimentally or clinically validated physiological phenomenon. Likewise, the absolute force values and percentages predicted for the implant-supported models cannot currently be considered quantitatively validated. The comparison with intact dentition therefore provides a calibrated physiological reference for evaluating relative changes in the numerical models. Still, it does not constitute independent validation of the implant-supported predictions. Future studies should incorporate independent experimental or clinical measurements of occlusal force distribution in implant-supported rehabilitations, together with sensitivity analyses over physiologically reasonable ranges of contact stiffness, to determine the quantitative accuracy and robustness of the predicted redistribution.

Mucosal representation is another relevant simplification. In the present model, the prosthesis–mucosa–bone interaction was reproduced using a bilinear pressure–overclosure law intended to approximate initial soft-tissue compression followed by stiffer contact with the underlying bone. Although this formulation captures the difference in compliance between the mucosa and the bone, it does not reproduce the non-linear, viscoelastic, time-dependent, and patient-specific behavior of oral mucosa, including stress relaxation, hysteresis, and variations in thickness. Since mucosal deformation affects prosthesis displacement and tilting, this approximation may modify load sharing between the soft tissue and the implants and, consequently, the predicted occlusal force distribution. Therefore, the specific 71–78% load concentration should not be treated as independent of the assumed mucosal behavior, and its magnitude requires confirmation using more realistic constitutive models and experimental data.

Finally, although the study included rehabilitations with zygomatic implants, it did not evaluate all surgical and prosthetic configurations described in the literature. Furthermore, the three analyzed configurations differ simultaneously in implant number, position, and type. Therefore, the observed differences cannot be attributed independently to conventional or zygomatic implants but must be interpreted as differences between complete rehabilitation configurations. Differences in implant number, inclination, bone quality, and behavior, as well as prosthetic design, could modify biomechanical behavior [2,3,6].

Future studies should therefore extend the present numerical framework to different bone qualities and more realistic, patient-specific material properties and should incorporate asymmetric and unilateral biting, oblique and lateral loading, and dynamic masticatory simulations. Controlled parametric analyses would also be required to separate, where anatomically meaningful, the contributions of implant number, position, and anchorage type. Sensitivity analyses of the occlusal contact parameters and more realistic representations of the implant–bone and prosthesis–mucosa interfaces would help to determine the robustness of the predicted force redistribution. Most importantly, independent experimental or clinical measurements of occlusal force distribution in implant-supported rehabilitations will be required to quantitatively validate the model predictions and determine whether the redistribution pattern identified under the present static symmetric condition occurs under actual functional conditions. Future work should analyze the temporal evolution of peri-implant bone remodeling and validate the results through clinical studies that allow the computational findings to be correlated with functional behavior.

## 5. Conclusions

The modeling assumptions adopted in this study proved suitable for simulating biomechanical behavior. Within the assumptions and loading conditions of the present finite element model, implant-supported prosthetic rehabilitation produced a marked model-predicted redistribution of posterior occlusal reaction forces compared with the physiological reference distribution for intact dentition. Under the investigated single static and symmetric bite condition, corresponding to a total reference occlusal force of 220.7 N, the model predicted that between 71% and 78% of the analyzed posterior occlusal force was concentrated at the first molar pair (46–36) in all three simulated rehabilitation configurations (71.1%, 77.6%, and 77.0% in simulated rehabilitation configurations 1, 2, and 3, respectively), whereas the physiological reference distribution showed the highest load at the second molar pair (47–37).

Within the adopted numerical framework, this redistribution reflects the combined mechanical response of the prosthesis, implant anchorage, soft-tissue support, and occlusal interaction. However, although the reference model was calibrated against occlusal force-distribution data from intact dentition, none of the three implant-supported configurations was independently validated against experimental or clinical force-distribution measurements. Therefore, the predicted 71–78% concentration at the 46–36 pair should be regarded as a model-dependent numerical prediction under the specific investigated assumptions and loading conditions rather than as an experimentally or clinically validated physiological phenomenon or a quantitatively established clinical value.

Among the three simulated configurations, simulated rehabilitation configuration 1, supported by three conventional implants per side, showed the most uniform posterior occlusal force distribution. This result must be attributed to the rehabilitation configuration as a whole rather than to implant type because the three cases differ simultaneously in the number of anchorages (six in simulated rehabilitation configuration 1 versus four in simulated rehabilitation configurations 2 and 3), their positions along the arch, and the type of anchorage. The present study design therefore does not allow for isolation of the independent effect of conventional versus zygomatic implants.

Under the type II bone conditions represented in the model, the three rehabilitation configurations showed only limited differences in the global stress state of the maxillary and zygomatic bones. This finding is specific to the favorable simulated bone quality. It should not be extrapolated to severely atrophic maxillae or to type III and IV bone, in which different biomechanical responses may occur.

Importantly, these findings are restricted to the single investigated static, symmetric loading condition and should not be interpreted as a general prediction of occlusal force distribution during functional mastication. The model did not reproduce asymmetric or unilateral biting, oblique or lateral force components, dynamic masticatory loading, or parafunctional conditions. Consequently, the present simulations cannot determine whether the predicted concentration of occlusal force at the 46–36 pair persists, decreases, increases, or shifts toward other contacts under these more complex loading conditions. Further numerical studies incorporating more clinically representative functional loading conditions, along with independent experimental or clinical force-distribution measurements in implant-supported rehabilitations, are needed to assess the robustness and quantitative validity of the predicted redistribution before extrapolating it to in vivo masticatory behavior. Limitations of the preferential load transmission study include the fact that all simulations used the same type II bone quality according to the Misch classification, which is considered favorable; behavior may differ in poorer bone-quality conditions. The present simulations cannot.

## Figures and Tables

**Table 1:** Input data used in the numerical models for the considered bone type (type II) [14].

	Young’s Modulus (GPa)	Poisson’s Ratio	Density (g/cm^3^)
Cancellous bone	5.5	0.3	2.12
Cortical bone	13.7	0.3	2.12

**Table 2:** Occlusal forces per tooth pair for a total load of 220.7 N.

Tooth Pair	Load (%)	Force per Tooth (N)
47–37	53.7%	59.3
46–36	31.2%	34.4
45–35	15.1%	16.7

**Table 3:** Comparison of the occlusal forces per tooth pair obtained from the FE models with the experimental reference values, for a total load of 220.7 N.

Tooth Pair	Test [10]	FE Simulated Rehabilitation Configuration 1	FE Simulated Rehabilitation Configuration 2	FE Simulated Rehabilitation Configuration 3
47–37	59.3 N	17.9 N	19.4 N	19.8 N
46–36	34.4 N	78.5 N	85.7 N	84.9 N
45–35	16.7 N	14.0 N	5.3 N	5.6 N

## Data Availability

The raw data supporting the conclusions of this article will be made available by the authors upon request.
